# Characteristics of second primary breast cancer after ovarian cancer: a Korea central cancer registry retrospective study

**DOI:** 10.3389/fonc.2023.1208320

**Published:** 2023-09-14

**Authors:** Eun-Gyeong Lee, Jiwon Lim, Hyeong In Ha, Myong Cheol Lim, Yoon Jung Chang, Young-Joo Won, So-Youn Jung

**Affiliations:** ^1^ Center for Breast Cancer, Research Institute and Hospital, National Cancer Center, Goyang, Republic of Korea; ^2^ Division of Cancer Registration and Surveillance, National Cancer Control Institute, National Cancer Center, Goyang, Republic of Korea; ^3^ Department of Obstetrics and Gynecology, Pusan National University Yangsan Hospital, Pusan National University School of Medicine, Yangsan, Republic of Korea; ^4^ Center for Gynecologic Cancer, Research Institute and Hospital, National Cancer Center, Goyang, Republic of Korea; ^5^ Department of Cancer Control and Population Health, National Cancer Center Graduate School of Cancer Science and Policy, Goyang, Republic of Korea; ^6^ Division of Tumor Immunology, Research Institute, National Cancer Center, Goyang, Republic of Korea; ^7^ Division of Cancer Control & Policy, National Cancer Control Institute, National Cancer Center, Goyang, Republic of Korea; ^8^ Division of Health Administration, Yonsei University, Wonju, Republic of Korea; ^9^ Cancer Healthcare Research Branch, Research Institute, National Cancer Center, Goyang, Republic of Korea

**Keywords:** ovarian cancer, breast cancer, second primary, survival, incidence

## Abstract

**Background:**

Second primary cancer has become an important issue among cancer survivors. This study sought to determine the differences in clinicopathologic outcomes between second primary breast cancer (SPBC) after ovarian cancer and primary breast cancer (PBC) in the Republic of Korea.

**Methods and materials:**

We searched the Korea Central Cancer Registry and identified 251,244 breast cancer cases that were diagnosed between 1999 and 2017. The incident rate and standardized incidence ratio (SIR) were calculated. Demographic and clinical characteristics and overall survival (OS) rates were estimated according to age, histological type, and cancer stage.

**Results:**

Among the 228,329 patients included, 228,148 were patients with PBC, and 181 patients had SPBC diagnosed after ovarian cancer (OC). The mean ages at diagnosis were 56.09 ± 10.81 years for SPBC and 50.65 ± 11.40 years for PBC. Patients with SPBC were significantly less likely than patients with PBC to receive adjuvant radiotherapy (14.92% vs. 21.92%, *p* = 0.02) or adjuvant chemotherapy (44.75% vs. 55.69%, *p* < 0.01). Based on the age-standardized rate (ASR), the incidence of SPBC after OC was 293.58 per 100,000 ovarian cancer patients and the incidence of PBC was 39.13 per 100,000 women. The SIR for SPBC was 1.27 (1.09-1.46, 95% Confidence interval) in the patients overall. The 5-year OS rates were 72.88% and 89.37% for SPBC and PBC (*p* < 0.01). The OS rate in SPBC decreased significantly with advanced stage and older age.

**Conclusion:**

The incidence of breast cancer is about 1.27 times higher in ovarian cancer patients than in healthy people. The survival outcomes were worse for SPBC than for PBC and were related to older age and advanced stage. Active screening for breast cancer is necessary in ovarian cancer patients.

## 1 Introduction

Breast cancer is the most common female cancer and it is steadily increasing worldwide (1). On the other hand, ovarian cancer has the highest mortality rate among gynecologic malignancies (2). The Republic of Korea has followed the global trend in this respect. According to 2016 nationwide cancer statistics in The Republic of Korea, breast cancer had the highest incidence in women, with a crude incidence rate of 85.0 per 100,000; additionally, about 2,600 people were newly diagnosed with ovarian cancer, which caused an estimated 1,200 deaths in 2016 (3).

Ovarian cancer ranks ninth in terms of cancer-related mortality, and in the Republic of Korea, the 5-year relative survival rate for patients with ovarian cancer stands at 58.1% (4). In 2020, the age-standardized cancer incidence rate for breast cancer was 61.0, while for ovarian cancer, it was 7.0 in the Republic of Korea. In terms of age-standardized cancer mortality, breast cancer recorded a rate of 6.1, whereas ovarian cancer had a rate of 2.5 (5). As an additional treatment option, the inclusion of olaparib maintenance therapy demonstrated a significant advantage in terms of progression-free survival for women who were diagnosed with newly advanced ovarian cancer and carried a *BRCA1/2* mutation (6). For individuals diagnosed with BRCA-mutated HER2-negative high-risk early breast cancer and metastatic breast cancer, olaparib can be considered as an adjuvant therapy choice subsequent to neoadjuvant chemotherapy or adjuvant chemotherapy (7, 8).

Survivors of primary cancer are increasing with advances in diagnostic methods and treatments. Consequently, second primary cancer has become an important issue among survivors. Various studies on second primary breast cancer following ovarian cancer have reported that this cancer is related to genetic factors, such as *BRCA* mutations (9–11). For these patients, the risk of breast cancer was reduced by treatment of ovarian cancer, which consists of salpingo-oophorectomy and platinum-based chemotherapy (12, 13). Some studies have reported differences in the clinical and survival outcomes and the risk of second primary breast cancer (14–16). However, these have mainly originated from western countries, and reports from Asian countries have been limited.

This study aimed to determine the risk and survival outcomes for patients with second primary breast cancer (SPBC) diagnosed after ovarian cancer. We also aimed to identify differences in clinicopathologic outcomes between second primary breast cancer and primary breast cancer (PBC).

## 2 Material and methods

### 2.1 Study population

Using the population-based Korea Central Cancer Registry (KCCR) database, we searched all patients with breast cancer who were diagnosed with PBC and SPBC after ovarian cancer between 1999 and 2017. We excluded breast cancer with other secondary cancers including endometrial cancer, lymphoma, colon cancer, thyroid cancer, etc. from the analysis. The KCCR was established in 1999 and is a representative population registry that collects about 98% of cancer data in the Republic of Korea.

The different types of breast cancer (C50) and ovarian cancer included the following: primary peritoneal cancer (C48), epithelial ovarian cancer (C56.9), and fallopian tube cancer (C57) which were all defined based on the basis of the International Classification of Diseases for Oncology, 3rd edition (ICD-O-3) (17).

Patients were grouped according to age at 10-year intervals (< 30 years, 30–39 years, 40–49 years, 50–59 years, 60–69 years, 70–79 years, and ≥ 80 years), date of diagnosis; Surveillance, Epidemiology, and End Results (SEER) stage; and the course of treatment, such as operation, radiation therapy, and chemotherapy within 4 months after the diagnosis of breast cancer.

### 2.2 Statistical analysis

The age-standardized rate (ASR) was calculated in patients diagnosed with breast cancer, using Segi’s world standard population and was expressed per 100,000 people. The standardized incidence ratio (SIR) of subsequent breast cancer among patients with ovarian cancer was calculated to quantify relative risk compared to the general population. We estimated cancer incidence for each cancer type according to age at diagnosis, latency, and diagnostic year, which was multiplied by the cumulative number of years at risk to calculate the number of cancers expected for each stratum. SIR was obtained by dividing the observed number of SPBCs in patients with ovarian cancer by the number of patients at risk of developing a new cancer in the general population (18). The 95% confidence intervals for the SIRs were estimated using Byar’s exact approximation to the accurate Poisson distribution of the observed number (19). The person-years at risk were calculated from two months after the initial ovarian cancer diagnosis until the date of the last known survival status, death, or study completion (December 31st, 2017).

Survival curves were created using the Kaplan-Meier method (20) and compared using the log-rank test, according to age, histological type, and SEER stage. All statistical tests were considered statistically significant at *p*-values of < 0.05. To compute SIR, “MP-SIR” session of SEER*Stat (version 8.3.6; National Cancer Institute, Bethesda, MD, USA) was used. Survival and other analyses were performed using SAS software (version 9.4; SAS Institute, Inc., Cary, NC, USA) and STATA software (version 16; StataCorp LLC, College Station, TX, USA).

## 3 Results

### 3.1 Characteristics of second primary breast cancer after ovarian cancer

According to the KCCR database, 251,244 patients were newly diagnosed with breast cancer from 1999 to 2017, of whom 228,148 were PBC patients and 181 were patients with SPBC diagnosed with ovarian cancer. As shown in **Table 1**, the ASR for breast cancer was 39.13per 100,000 women. The ASR of SPBC was 0.03 per 100,000 women and 293.58 per 100,000 after ovarian patients.

**Table 1:** Age-Standardized Rate of breast cancer by age, 1999-2017.

Age(yrs)	Breast cancer			Second primary breast cancer after ovarian cancer			
	ASR* per 100,000 women	% of cases	Cases	ASR* per 100,000 women	ASR per 100,000 ovarian cancer patients	% of cases	Cases
<30	1.09	1.36	3418	0.00	44.13	0.55	1
30-39	4.94	12.81	32181	0.00	44.61	5.52	10
40-49	14.21	36.41	91490	0.01	69.80	24.31	44
50-59	10.69	27.76	69736	0.01	60.80	32.60	59
60-69	6.12	13.96	35069	0.01	53.69	25.41	46
70-79	1.74	6.06	15216	0.00	15.09	9.39	17
≥80	0.36	1.65	4134	0.00	5.46	2.21	4
Total	39.13	100.00	251244	0.03	293.58	100.00	181

*ASR (Age-Standardized Rate) was calculated using Segi’s world standard population; yrs, years.

**Table 2** shows the clinicopathological characteristics of patients with breast cancer. The mean age at diagnosis of patients with SPBC after ovarian cancer was 56.09 ± 10.81 years and that of patients with PBC was 50.65 ± 11.40 years. The SPBC was diagnosed at a significantly older age than the PBC (*p* < 0.01). The most common age group at SPBC diagnosis was 50–59 years (32.60%), while the most common age group for diagnosis of PBC was 40–49 years (37.07%, *p* < 0.01). Both groups were most frequently diagnosed with local stage (65.71% of SPBC vs. 55.70% of PBC cases, *p* = 0.18) and the invasive carcinoma of no special type (NST) was the most common histological type (84.53% of SPBC vs. 88.98% of PBC cases, *p* = 0.01). Surgery was performed in 82.87% of SPBC cases and 86.94% of PBC cases (*p* = 0.11).

**Table 2:** Clinicopathologic characteristics of patients with breast cancer in the Republic of Korea, 1999-2017.

Characteristic	SIR¶	CI	Observed	Expected	Incidence per 100,000 person-years	Second primary breast cancer after ovarian cancer	primary breast cancer	P-value**
**Cases (Total patients)**	1.27*	1.09-1.46	181	143.04	13.35	181(178)	228148	–
**Follow-up (yr.): Median (Range)**						9.00 (0.58-18.67)	6.38 (4.84)	<0.01
Follow-up from second primary breast cancer diagnosis (yr): Median (Range)	–					2.51 (0.02-15.99)	–	–
**Age at breast cancer diagnosis: Mean (SD)**	–					56.09 (10.81)	50.65 (11.40)	<0.01
Age at diagnosis of ovarian cancer (yr.): Mean (SD)	–					51.17 (10.14)	–	–
Age group at ovarian cancer diagnosis (yr.)								
<30	3.71	0.09-20.68	1	0.27	2.11	1 (0.55%)	3235 (1.42%)	<0.01
30-39	1.73	0.83-3.18	10	5.79	8.34	10 (5.52%)	30306 (13.28%)	
40-49	1.12	0.81-1.50	44	39.33	15.30	44 (24.31%)	84582 (37.07%)	
50-59	1.06	0.81-1.37	59	55.58	13.95	59 (32.60%)	63006 (27.62%)	
60-69	1.53*	1.12-2.04	46	30.00	15.79	46 (25.41%)	30595(13.41%)	
70-79	1.60	0.93-2.55	17	10.65	11.17	17 (9.39%)	13126 (5.75%)	
≥ 80	2.84	0.77-7.26	4	1.41	11.50	4 (2.21%)	3298 (1.45%)	
Histologic type								
NST^c^	1.21*	1.02-1.41	153	126.84	11.28	153 (84.53%)	203006 (88.98%)	0.01
Lobular	0.86	0.28-2.01	5	5.80	0.37	5 (2.79%)	8334 (3.65%)	
Mucinous	0.71	0.09-2.58	2	2.80	0.15	2 (1.1%)	4353 (1.91%)	
Medullary	3.58	0.74-10.47	3	0.84	0.22	3 (1.66%)	1894 (0.83%)	
Metaplastic	0.97	0.02-5.38	1	1.04	0.07	1 (0.55%)	1424 (0.62%)	
Papillary	4.36*	1.60-9.50	6	1.37	0.44	6 (3.31%)	2408 (1.06%)	
Others	2.53*	1.26-4.52	11	4.35	0.81	11 (6.08%)	6729 (2.95%)	
Stage at diagnosis (Since 2006)								
Local	1.34*	1.04-1.69	69	51.53	9.20	69 (65.71%)	99481 (55.70%)	0.18
Regional	0.95	0.63-1.38	28	29.35	3.73	28 (26.67%)	60777 (34.03%)	
Distant	1.15	0.37-2.69	5	4.34	0.67	5 (4.76%)	8684 (4.86%)	
Unknown	0.91	0.19-2.66	3	3.29	0.40	3 (2.86%)	9675 (5.42%)	
Surgery								
Yes	1.22*	1.03-1.43	150	122.80	11.06	150 (82.87%)	198342 (86.94%)	0.11
No	1.53*	1.04-2.17	31	20.24	2.29	31 (17.13%)	29806 (13.06%)	
Radiation therapy								
Yes	0.92	0.61-1.34	27	29.32	1.99	27 (14.92%)	50010 (21.92%)	0.02
No	1.35*	1.15-1.59	154	113.72	11.36	154 (85.08%)	178138 (78.08%)	
Chemotherapy								
Yes	1.08	0.86-1.34	81	75.19	5.97	81 (44.75%)	127064 (55.69%)	<0.01
No	1.47*	1.20-1.79	100	67.85	7.38	100 (55.25%)	178138 (78.08%)	
Interval between 1st and 2nd cancers (yr.)								
Median (Range)	–					4.00 (0.17-17.50)	–	–
<1	1.05	0.65-1.61	21	19.98	10.38	21	–	–
1-4	1.34*	1.07-1.65	86	64.32	13.85	86	–	
5-9	1.31	0.98-1.73	50	38.05	14.19	50	–	
≥10	1.16	0.74-1.73	24	20.69	13.32	24	–	

¶SIR: Standardized Incidence Ratio ANOVA tests and chi-square tests were performed to evaluate differences by factor for continuous variables, and for categorical variables, respectively. ^c^ NST: No Special Type, CI: Confidence interval.

* Significant at alpha = 0.05. **means is ANOVA tests and chi-square tests were performed to evaluate differences by factor for continuous variables, and for categorical variables, respectively.

The SIR for SPBC was 1.27 in the patients overall, and 1.53 in women aged 60–69 years. The papillary type showed a significantly higher SIR (4.36). The SIR of the NST subtype was 1.21. When comparing the SIR according to surgical status, the SIR was significantly higher when surgery was not performed (SIR 1.22 with surgery vs. SIR 1.53 without surgery). In terms of additional treatment, the SIR was 1.35 without radiation therapy, and 1.47 without chemotherapy.

A total of 181 women diagnosed with SPBC were evaluated for a mean follow-up period of 8.95 years. The mean interval from the diagnosis of ovarian cancer to the diagnosis of SPBC was 5.05 years. In this group, the SIR between 1 and 4 years increased significantly to 1.34.

### 3.2 Survival outcomes

**Figure 1:**
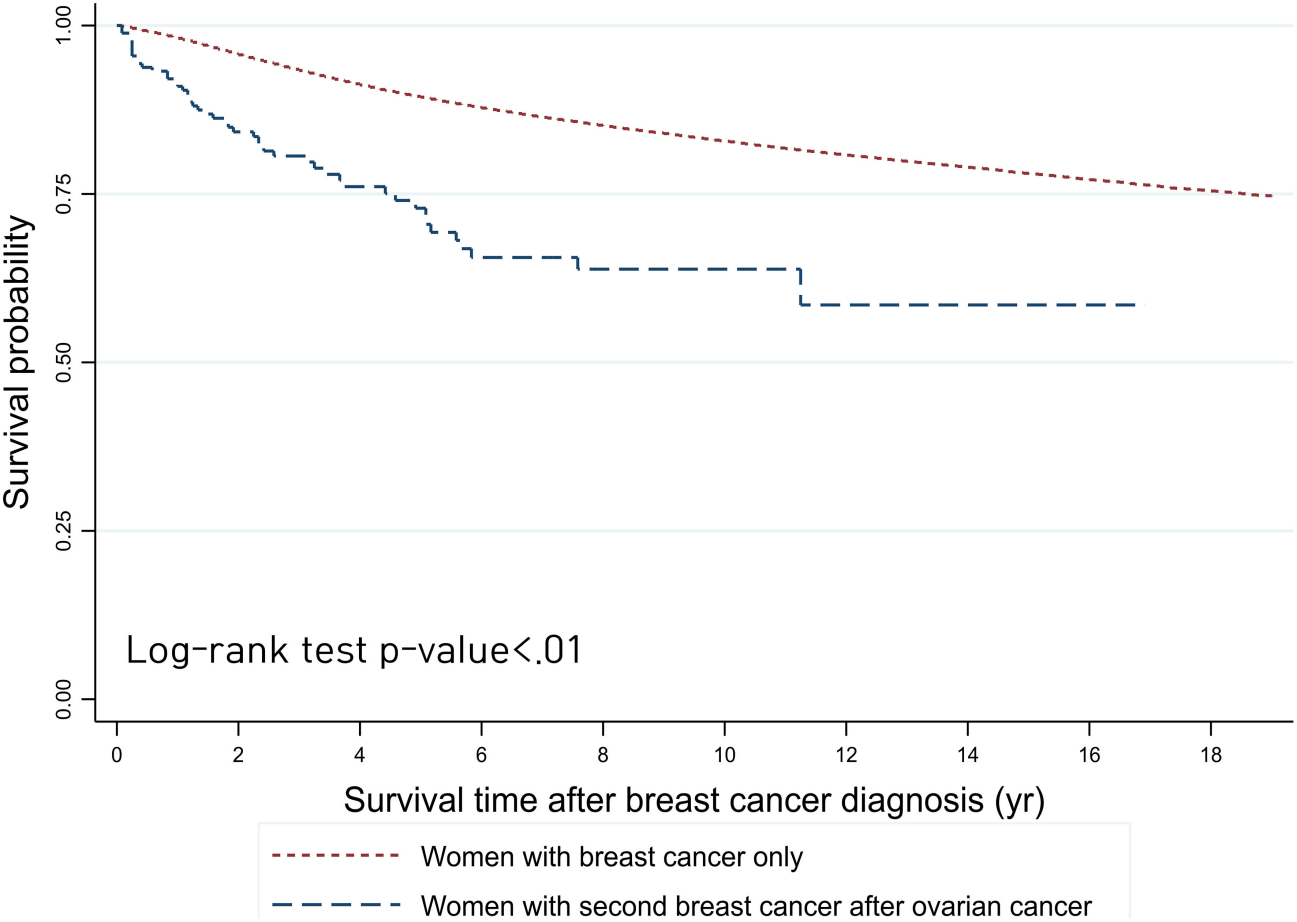
Survival outcomes of patients with breast cancer from the onset time of breast cancer diagnosis in the Republic of Korea.

**Figure 2:**
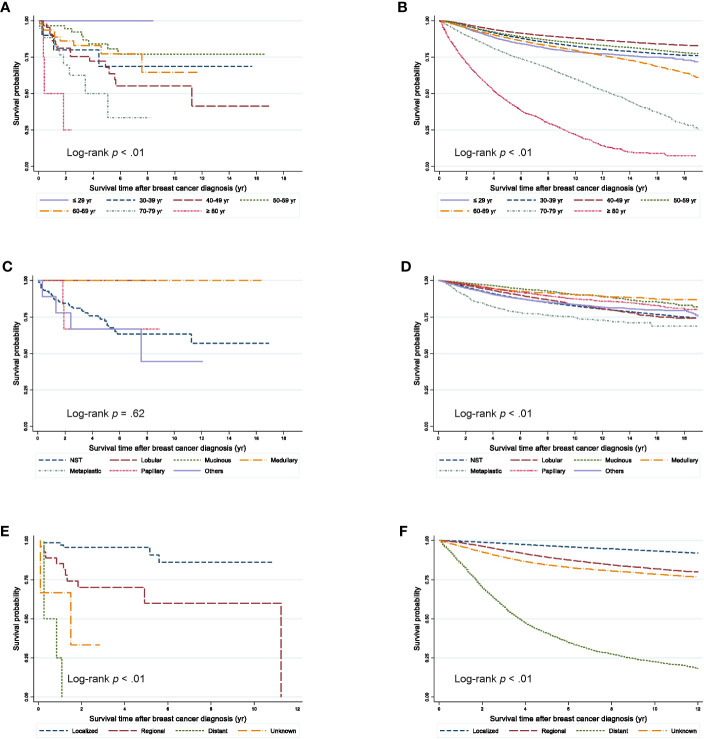
Survival outcomes from the onset time of breast cancer diagnosis according to clinicopathologic characteristics. **(A)** Second primary breast cancer after ovarian cancer according to age groups **(B)** Primary breast cancer according to age groups **(C)** Second primary breast cancer after ovarian cancer according to histological types. **(D)** Primary breast cancer according to histological types. **(E)** Second primary breast cancer after ovarian cancer according to the SEER stage since 2006. **(F)** Primary breast cancer according to the SEER stage since 2006.

The 5-year OS rate from the onset of SPBC was 72.88% and that from the onset of PBC was 89.37% (**Figure 1**). Compared by age, the 5-year OS rate of patients with SPBC was low, at 50.05%, in patients aged 70–79 years and the median survival time was 61 months (**Figure 2**). The 5-year OS rate of patients aged 40–49 years was also low, at 67.95%, and the median survival time was 135 months. For PBC, the 5-year OS rate was 44.43% in patients over 80 years of age, and the median survival time was 51 months. The 5-year OS rate was 77.21% in patients aged 70–79 years and the medial survival time was 151 months.

Among cases with NST histology, the 5-year OS rates were 72.07% in SPBC and 89.16% in PBC cases. When compared by stage, patients with SPBC had a significantly worse prognosis than those with PBC.

## 4 Discussion

In this study, we investigated differences in clinicopathologic outcomes between SPBC after ovarian cancer and PBC in the Republic of Korea. We showed that the incidence of breast cancer is higher in patients with ovarian cancer than it is in healthy people and that survival outcomes were worse for SPBC than for PBC; increased mortality was related to older age and advanced cancer stage.

In the current study, SPBC after ovarian cancer accounted for only 0.07% of newly diagnosed breast cancers in the Republic of Korea, based on KCCR data from a 19-year period. In a retrospective observational study using SEER data between 1973 and 2013, the incidence of SPBC in the ovarian cancer cohort was approximately 1.37% (1,821/133,149) and the incidence of SPBC decreased from 7.2% to 2.0% between 1973 and 2008 (*P* < 0.05) (21). From 1999 to 2017, the incidence of breast cancer continued to increase year by year. On the other hand, the incidence of SPBC after ovarian cancer did not remain constant. In this study, the incidence of SPBC after ovarian cancer increased about two-fold in 2016 and 2017 (Supplement 1). Increasing rates of SPBC may be due to interest in genetic testing and application of insurance reimbursement, which may have led to active screening for breast cancer.

Ovarian cancer and breast cancer are hereditary cancers, and *BRCA1* and *BRCA2* are well known as causative genes (9). According to the National Comprehensive Cancer Network guideline, risk-reducing surgery is recommended for carriers of *BRCA* gene mutations at an appropriate time (12). A previous meta-analysis reported that the risk of breast cancer was reduced by about 50% in carriers of *BRCA1* and *BRCA2* mutations who underwent risk-reducing bilateral salpingo-oophorectomy (RRSO) (13). In our previous study about patterns of risk-reducing surgery in carriers of *BRCA* mutations, the rate of RRSO was 42.5%. After insurance reimbursement started in 2013, the rate of risk-reducing surgery was significantly higher (46.3% after 2013 vs. 31.6% before 2013, *p* < 0.001) (22).

In the current study, the overall SIR for SPBC after ovarian cancer was 1.27 (95% CI, 1.09–1.46). In a study of invasive ovarian cancer, the SIR of breast cancer was 1.35 (95% CI, 0.85–2.05) (15). In another study on an ovarian cancer cohort from the Stockholm–Gotland Cancer Registry, the SIR of breast cancer was 1.41 (95% CI, 1.14–1.75) (14). We found that the SIR of SPBCS was significantly higher at 1.34 between 1 and 4 years after the initial diagnosis of ovarian cancer. Thus, patients with ovarian cancer require more active surveillance for SPBC during this period.

This study showed that patients with SPBC after ovarian cancer had a markedly poor survival. Interestingly, when compared by age, our study confirmed that the survival rate of patients with PBC was relatively lower in the older age-group, whereas the survival rate of patients with SPBC was lower in the 40s age-group. Treatment of ovarian cancer with radiation therapy and chemotherapy may lead to aggressiveness of the second primary tumor. A previous study using the SEER program database showed that survival of patients with localized SPBC was similar to that of patients with PBC, while cases with advanced stage SPBC showed worse survival outcomes (16). Our study showed similar results.

This study had several strengths. We analyzed a population-based cancer registry that included approximately 98% of Korean cancer cases. No previous study has reported the clinical outcomes of patients with breast cancer after ovarian cancer in the Republic of Korea. However, our study had several limitations. First, this study lacked detailed clinical information regarding surgery type, such as mastectomy or breast-conserving surgery, and subtypes of tumor, such as luminal A, luminal B, HER2-positive, and triple-negative. Second, the study did not have access to personal history, family history, and status of genetic mutations, such as those in *BRCA1/BRCA2*, which influence hereditary cancer. Especially, *BRCA1/BRCA2* mutation carriers have an increased risk for breast and ovarian cancer. *BRCA1* was found to be linked with an elevated risk across all subtypes in breast cancer, although the odds ratios (ORs) exhibited significant variation, with the highest OR observed in triple-negative disease (OR, 55.32; 95% CI, 40.51-75.55). Additionally, *BRCA2* and *PALB2* variants were also associated with triple-negative (23). By the age of 70, individuals with *BRCA1* mutations had an estimated breast cancer penetrance of 64.6% (95% CI = 59.5% to 69.4%), while those with *BRCA2* mutations had a penetrance of 61.0% (95% CI = 48.1% to 72.5%). As for ovarian cancer, the estimated penetrance by age 70 was 48.3% (95% CI = 38.8% to 57.9%) for *BRCA1* mutation carriers and 20.0% (95% CI = 13.3% to 29.0%) for *BRCA2* mutation carriers (24). In this study, patients with second primary breast cancer were observed to have a lower likelihood of receiving adjuvant radiotherapy and chemotherapy compared to those with primary breast cancer. This discrepancy could be attributed to factors such as the stage of ovarian cancer and the treatment options for cancer. However, it is important to acknowledge that this limitation arises due to the nature of national cohort data, which does not provide confirmatory information on treatment options or stages of ovarian cancer. This study could indirectly determine a patient’s condition for each cancer, but could not describe this with limited data on treatment history and causes of death. Further studies should perform detailed investigations regarding the breast cancer characteristics.

In conclusions, the present study confirmed that the incidence of SPBC in patients with ovarian cancer was higher than that observed in those with primary breast cancer, and the survival outcome was low. However, if it the cancer is detected in the early stages, it shows a survival rate similar to those with PBC. Hence, active screening for breast cancer is necessary in patients with ovarian cancer between 1 and 4 years after diagnosis of ovarian cancer.

## Data availability statement

The raw data supporting the conclusions of this article will be made available by the authors, without undue reservation.

## Ethics statement

All procedures performed in studies involving human participants were in accordance with the ethical standards of the institutional and/or national research committee and with the 1964 Helsinki Declaration and its later amendments. The requirement for obtaining informed patient consent was waived because this was a secondary analysis of de-identified data and this study was approved by the Institutional Review Board of the National Cancer Center, The Republic of Korea (IRB number: NCC2020-0195). No administrative permission and/or licenses is acquired by this study to access the original data used in this research.

## Author contributions

E-GL, ML, Y-JW, S-YJ: conceptualization. Y-JW, JL: data curation. Y-JW, JL: formal analysis. E-GL, Y-JW, ML, S-YJ, JL: investigation. Y-JW, JL: methodology. S-YJ: project administration. Y-JW, ML, S-YJ, E-GL: funding acquisition. E-GL: writing the original draft. E-GL, Y-JW, ML, S-YJ, HH, Y-JC: writhing review & editing. Y-JW, S-YJ: supervision. All authors contributed to the article and approved the submitted version.
