# Supplementary material for: Characteristics of second primary breast cancer after ovarian cancer: a Korea central cancer registry retrospective study
Source: Front Oncol. 2023 Sep 14;13:1208320. doi: 10.3389/fonc.2023.1208320 (PMC10539581; doi:10.3389/fonc.2023.1208320)
Supplement: Supplementary file 1 [file Table_1.docx]

Supplementary Material

Characteristics of second primary breast cancer after ovarian cancer: A Korea central cancer registry retrospective study

Eun-Gyeong Lee*, Jiwon Lim, Hyeong In Ha, Myong Cheol Lim, Yoon Jung Chang, Young-Joo Won, So-Youn Jung

*** Correspondence:** Young-Joo Won: youngwon@yonsei.ac.kr, So-Youn Jung: goje1@ncc.re.kr

# Supplementary Table

Supplementary TABLE 1. Incidence of breast cancer by year of diagnosis, 1999-2017

| Year | Breast cancer | | | Second primary breast cancer  after ovarian cancer | | |
| --- | --- | --- | --- | --- | --- | --- |
|  | ASR*  per 100,000  women | % of cases | Cases | ASR  per 100,000  ovarian patients | % of cases | Cases |
| 1999 | 21.37 | 2.32 | 5824 | 35.57 | 0.55 | 1 |
| 2000 | 21.53 | 2.39 | 6010 | 74.52 | 1.10 | 2 |
| 2001 | 25.25 | 2.90 | 7277 | 77.44 | 1.10 | 2 |
| 2002 | 27.83 | 3.27 | 8225 | 155.45 | 2.21 | 4 |
| 2003 | 27.95 | 3.37 | 8474 | 172.21 | 1.66 | 3 |
| 2004 | 29.50 | 3.65 | 9175 | 187.41 | 2.21 | 4 |
| 2005 | 32.32 | 4.07 | 10236 | 109.47 | 2.21 | 4 |
| 2006 | 33.64 | 4.34 | 10898 | 384.91 | 6.08 | 11 |
| 2007 | 36.17 | 4.77 | 11987 | 45.16 | 1.10 | 2 |
| 2008 | 37.77 | 5.09 | 12791 | 223.95 | 4.97 | 9 |
| 2009 | 39.32 | 5.42 | 13628 | 368.76 | 7.18 | 13 |
| 2010 | 41.38 | 5.83 | 14636 | 998.19 | 6.08 | 11 |
| 2011 | 44.58 | 6.42 | 16133 | 297.86 | 7.73 | 14 |
| 2012 | 45.39 | 6.66 | 16728 | 411.47 | 6.63 | 12 |
| 2013 | 46.34 | 6.94 | 17432 | 148.82 | 4.42 | 8 |
| 2014 | 48.02 | 7.34 | 18436 | 322.07 | 8.29 | 15 |
| 2015 | 49.59 | 7.66 | 19254 | 209.80 | 7.18 | 13 |
| 2016 | 55.41 | 8.68 | 21805 | 337.88 | 14.36 | 26 |
| 2017 | 56.07 | 8.87 | 22295 | 523.48 | 14.92 | 27 |
| 1999-2017 | 39.13 | 100.00 | 251244 | 293.58 | 100.00 | 181 |

* ASR (Age-Standardized Rate) was calculated using Segi's world standard population.
